# Transfixing Kirshner wires for fixation of intertrochanteric valgus osteotomies in management of pediatric coxa vara

**DOI:** 10.1007/s10195-017-0463-x

**Published:** 2017-07-12

**Authors:** Ahmed Shawkat Rizk

**Affiliations:** Orthopaedics and Traumatology Department, Benha Faculty of Medicine, Benha University, Farid Nada Street, Benha, Qualiobia Egypt

**Keywords:** Pediatric coxa vara, Valgus osteotomy, Transfixing wires, Satisfactory results, Simple surgical technique

## Abstract

**Background:**

Coxa vara is a radiological term describing a decrease in the neck–shaft angle to 120° or less. Coxa vara is associated with pathomechanical changes that can manifest clinically. If left untreated, coxa vara can affect the normal development of the pediatric hip. Valgus osteotomy is the standard surgical treatment for coxa vara, but there is no consensus regarding the optimal osteotomy technique and fixation method. The work reported here aimed to highlight transfixing wires as a fixation method for valgus osteotomy applied as treatment for various types of pediatric coxa vara.

**Materials and methods:**

This study included 16 cases of pediatric coxa vara with different etiologies in 9 patients with a mean age of 39.9 ± 15.2 months. Radiological and clinical evaluations and scoring of the condition of each patient according to the Iowa Hip Score were performed pre- and postoperatively. Transfixing wires and a protective spica were used for the fixation of a V-shaped, laterally based, closing-wedge valgus osteotomy in all cases. The postoperative follow-up period ranged from 14 to 102 months, with a mean duration of 33.3 ± 27.7 months.

**Results:**

The mean Hilgenreiner epiphyseal angle (HEA) was corrected from 81.7 ± 2.2° to 24.3 ± 3.5° and the mean femoral neck–shaft angle (FNSA) was improved from 86.9 ± 4.2° to 138.6 ± 3.5°. No recurrence of the deformity was observed during the follow-up periods considered here. The osteotomy site united after an average of 11.7 ± 2.2 weeks with no secondary displacement, and in cases of developmental coxa vara there was progressive ossification of the neck defect with no surgery-related complications. Clinical results were markedly improved by the osteotomy, with a mean postoperative Iowa Hip Score at last follow-up of 95.06 ± 2.6, compared to a mean preoperative score of 57.4 ± 3.6.

**Conclusions:**

Transfixing wires protected in a hip spica cast represent a simple, easy, and reliable fixation method for valgus osteotomies performed to correct pediatic coxa vara. It assures stable fixation and rapid healing of the osteotomy without loss of the achieved correction, it completely avoids the femoral neck affording marked protection to the growth plate.

**Level of evidence:**

IV.

## Introduction

The normal pediatric femoral neck–shaft angle (FNSA) ranges from 135° to 145°. Coxa vara is a condition in which the femoral neck–shaft angle on one side is decreased compared to that on the unaffected side in unilateral cases, or the neck–shaft angle is 120° or less in bilateral cases. Coxa vara is a relatively uncommon condition with an estimated incidence of 1 in 25,000 live births. It is equally likely to occur in either gender [1].

**Fig. 1:**
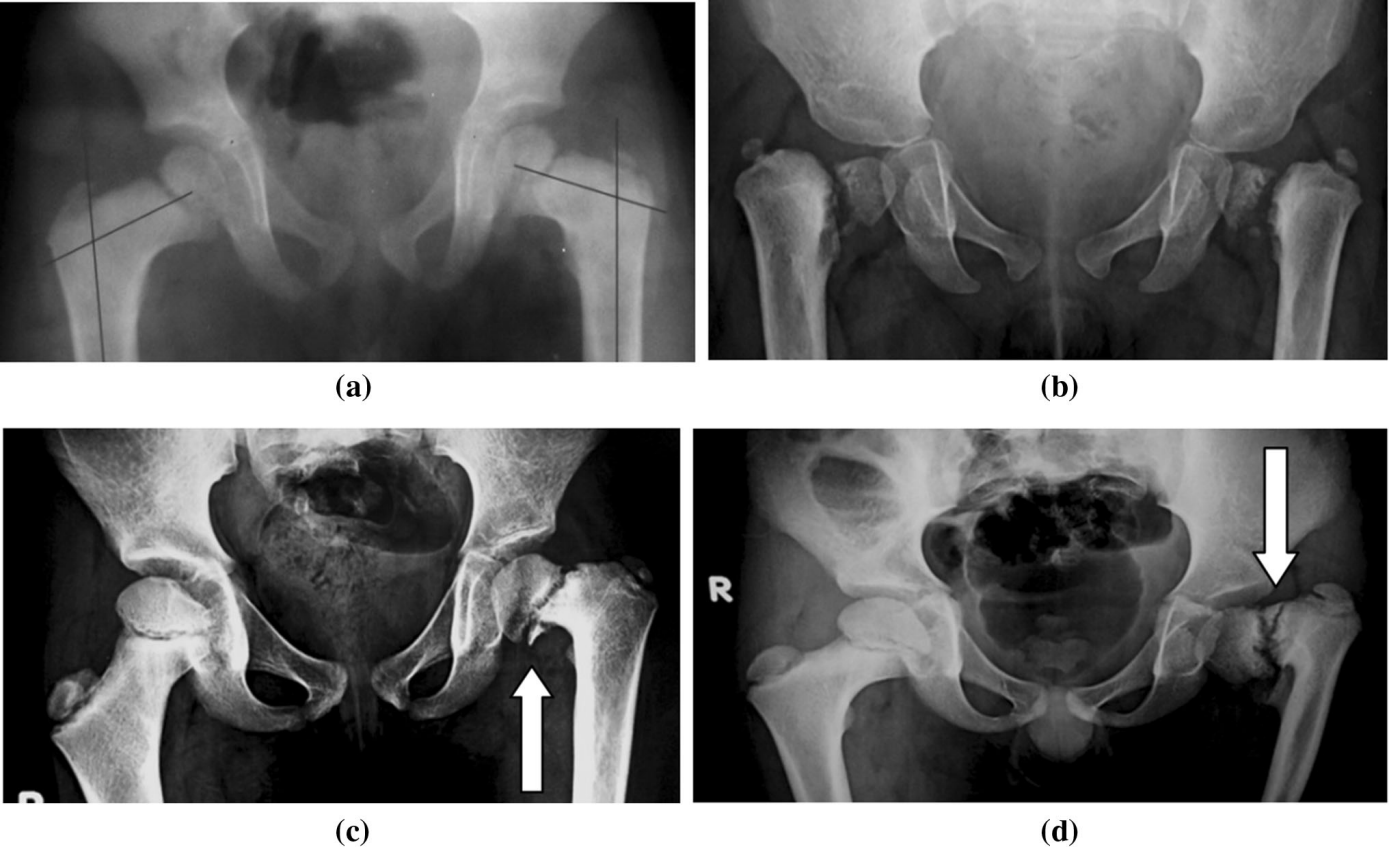
Standard AP radiographs of the pelvis and both hip joints showing different etiologies and severities of coxa vara. **a** Bilateral coxa vara following healed rickets. **b** Bilateral developmental coxa vara with a medial femoral neck defect and a vertically oriented physeal plate. **c** Typical unilateral coxa vara with a medial (lower) femoral neck defect. **d** Atypical unilateral coxa vara with a lateral (upper) femoral neck defect

Coxa vara is not a pathological diagnosis; it is a radiological term describing a deformed proximal femur that could result from different pathological conditions (Fig. 1). Coxa vara could follow healed rickets or other bone-softening diseases (Fig. 1a), could be developmental (Fig. 1b) or associated with congenital femoral deficiency or could be post-traumatic, but there is no universally accepted classification system for this condition [2].

Developmental coxa vara is a pathobiological condition characterized by an ossification defect typically affecting the medial portion of the femoral neck (Fig. 1c); however, in some rare cases, it can also affect the lateral portion of the femoral neck (Fig. 1d). Developmental coxa vara is associated with progressive pathomechanical changes affecting the proximal femur that—in addition to the clinical manifestations—can markedly affect the growing pediatric hip, leading to premature degenerative arthritis of the hip joint.

Once deformed, the hip joint is progressively subjected to bending stresses and shear forces, leading to a progressive decrease in the neck–shaft angle and a more vertically oriented proximal femoral physeal plate due to the bending stresses, as well as neck shortening and overgrowth of the greater trochanter [2, 3], thus triggering a vicious circle.

Indications for surgical correction are based on the clinical picture and the patient’s symptoms, the neck–shaft angle, and—more importantly—the Hilgenreiner epiphyseal angle (HEA) [3, 4]. The goal of surgical intervention is to correct varus malalignment of the neck, thereby changing the loading characteristics from shear to compression forces, which can stimulate ossification and healing of the defective femoral neck and subsequent normalization of the neck–shaft angle. Proximal femoral valgus osteotomy is considered the standard surgical treatment for coxa vara, and multiple surgical techniques with different fixation methods, including external fixators and internal fixation with pins, cerclage wiring, and a variety of plates, have been described [5].

## Materials and methods

### Clinical data

This prospective case series included 16 cases (Table 1) of pediatric coxa vara in nine patients (seven patients were affected bilaterally and two unilaterally), and was carried out in the orthopedics department of Benha University Hospital from February 2007 to October 2016. The study included six males and three female patients with ages at presentation ranging from 26 to 68 months (mean age: 39.9 ± 15.2 months). The patients had different etiologies and degrees of severity of coxa vara: two cases occurred following rickets, and there were 14 cases of developmental coxa vara. There was no previous surgical interference before presentation in 13 of the cases, but a previous bilateral adductor tenotomy was implemented 12 months before presentation in two cases (one patient) while the other case had a previous unsuccessful valgus osteotomy to correct for unilateral developmental coxa vara which was fixed by plate and screws.

**Table 1:** Characteristics of the studied cases

Parameters		Age (months)	Etiology	Previous surgery	Union time of the osteotomy (weeks)	Follow up period (months)	Comments
Patients	Cases						
1 (Male)	1	29	DCV	–	10	22	Gluteal heterotropic ossification
	2	29	DCV	–	10	22	–
2 (Male)	3	34	DCV	–	9	29	Medialization of the femur
	4	34	DCV	–	14	29	–
3 (Male)	5	31	DCV	Adductor tenotomy	13	15	–
	6	31	DCV	Adductor tenotomy	13	15	–
4 (Female)	7	44	DCV	Failed previous osteotomy	15	27	Medialization of the femur LLD 1 cm
5 (Male)	8	45	Healed rickets	–	11	102	Sunken wires
	9	45	Healed rickets	–	11	102	Sunken wires
6 (Male)	10	26	DCV	–	9	22	–
	11	26	DCV	–	9	22	–
7 (Male)	12	69	Atypical DCV	–	15	19	Premature closure of the epiphysis Medialization of the femur Gluteal heterotropic ossification.
8 (Female)	13	66	DCV	–	15	36	–
	14	66	DCV	–	14	36	–
9 (Female)	15	32	DCV	–	10	17	–
	16	32	DCV	–	10	17	–
Mean		39.9 ± 14.7			11.7 ± 2.2	33.3 ± 27.7	

Parameters									
Patients	Cases	Pre-op. IOWA score	Post-op. IOWA score	Pre-op. FNSA	Early post-op. FNSA	Last follow-up FNSA	Pre-op. HEA	Early post-op. HEA	Last follow-up HEA
1 (Male)	1	55	94	91	136	138	88	22	22
	2	55	94	91	135	138	88	24	22
2 (Male)	3	63	96	88	140	142	83	27	25
	4	60	95	89	139	139	84	26	22
3 (Male)	5	58	95	79	140	140	79	25	25
	6	58	95	79	140	140	80	27	27
4 (Female)	7	59	91	89	137	141	77	28	26
5 (Male)	8	60	95	117	132	135	61	23	20
	9	60	96	116	133	137	64	24	20
6 (Male)	10	53	97	88	134	134	88	27	27
	11	53	97	88	136	136	88	29	29
7 (Male)	12	62	96	86	130	130	87	26	Could not be accurately detected (premature physeal closure)
8 (Female)	13	52	96	76	142	144	85	23	21
	14	52	95	76	141	142	85	22	20
9 (Female)	15	58	97	90	141	141	84	29	29
	16	58	96	90	140	140	86	29	29
Mean		57.3 ± 3.5	95.3 ± 1.2^a^	89.7 ± 11.7	137.3 ± 3.7^b^	138.6 ± 3.5	81.6 ± 8.2	25.7 ± 2.5^c^	24.3 ± 3.5
P value		<0.001		<0.001			<0.001		
					NS			NS	

*FNSA* femoral neck shaft angle, *HEA* hilgenreiner-epiphyseal angle, *DCV* developmental coxa vara, *LLD* limp length discrepancy, *NS* non-significant difference between early post-op. and last follow-up FNSA and HEA angles

^a^Significant difference between post-op. IOWA score and pre-op. IOWA score

^b^Significant difference between early post-op. FNSA and pre-op. FNSA

^c^Significant difference between early post-op. HEA and pre-op. HEA

### Clinical evaluation

The preoperative evaluation focused on each patient’s complete history, physical examination, and Iowa Hip Score. The patients typically presented with painless and progressive limping caused by either a shortening of the affected side and/or the Trendelenburg gait in unilateral cases or a waddling gait with exaggerated lumbar lordosis and prominent greater trochanters in bilateral cases. A positive Trendelenburg test and a limb length discrepancy (LLD) of about 2 cm were characteristic of the unilateral cases. Restricted internal rotation and abduction of the affected hip—even under general anesthesia—are typical signs of coxa vara. Genu valgum was not present in any of the studied cases.

### Radiological evaluation

A standard AP radiograph of the pelvis and both hip joints (Fig. 1) was sufficient to diagnose coxa vara and to highlight the pathological changes in the affected hip joint (proximal femur and acetabulum). The most important radiological findings were:

- Varus alignment of the femoral neck with a FNSA of 120° or less
- Loss of the normal horizontal orientation of the physis (it had a more vertically oriented position) and widening of the physeal plate
- A relatively overgrown greater trochanter

**Fig. 2:**
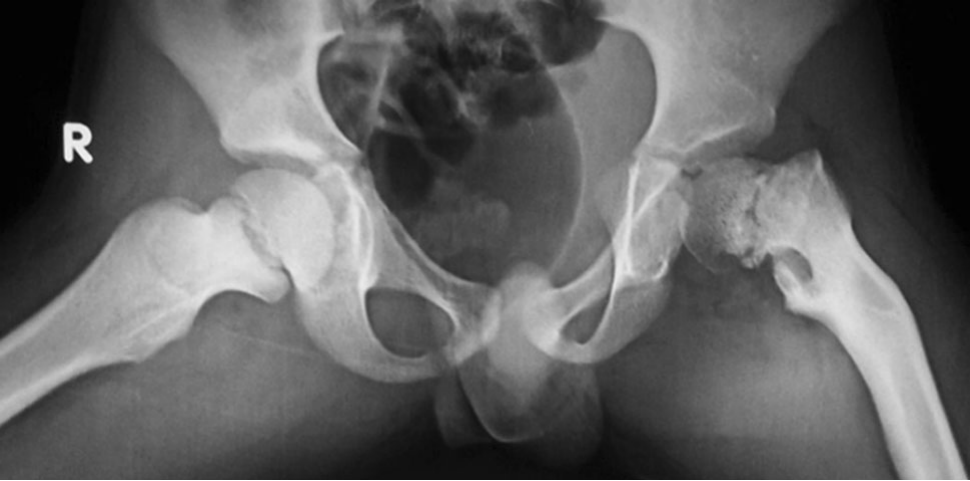
A standard frog-leg lateral radiograph of the pelvis and both hip joints showed a dysplastic proximal femur and acetabulum with a retroverted neck on the affected side

A standard frog-leg lateral radiograph of the pelvis and both hip joints (Fig. 2) was less informative and could only be used to discern neck version and dysplasia of the proximal femur. The neck was retroverted in the cases of developmental coxa vara, in contrast to the cases of coxa vara following healed rickets.

**Fig. 3:**
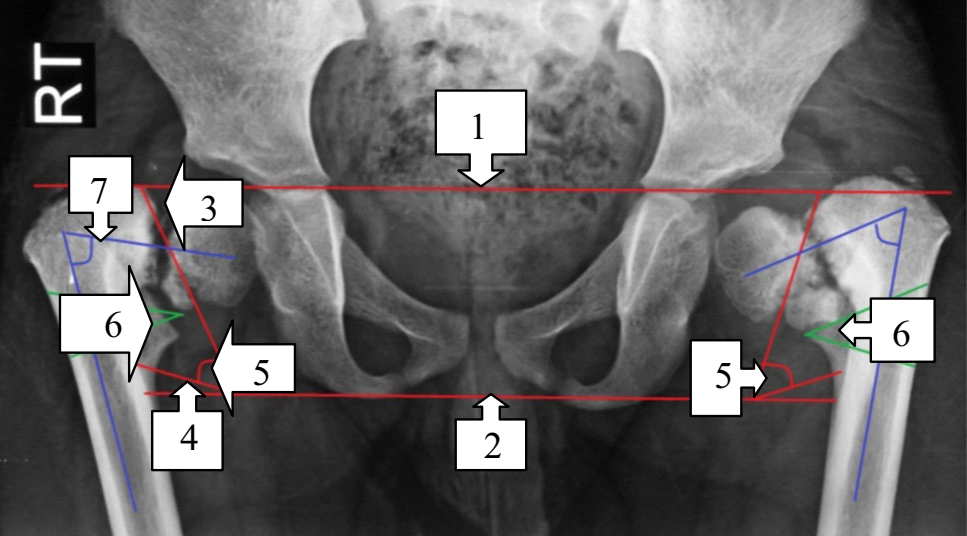
Analyzing the deformity and planning the procedure. *1* Hilgenreiner line, (line through both triradiate cartilages). *2* Interischial line. *3* Hilgenreiner epiphyseal angle (HEA). *4* The fixed angle (16°) that is subtracted from the Hilgenreiner epiphyseal angle (HEA). *5* The actual osteotomy angle (angle of correction); angle* 5* = angle* 3* − angle* 4*. *6* The* angle between the green lines* represents the actual angle of the corrective osteotomy; angle* 6* = angle* 5*. *7* *Line* bisecting the neck of the femur that is used when planning the upper line of the osteotomy

The standard AP radiograph was also used to analyze the deformity and to plan the procedure as follows (Fig. 3):

- The Hilgenreiner line (labeled 1 in Fig. 3) is used to calculate the Hilgenreiner epiphyseal angle (HEA; labeled 3 in Fig. 3) proximal to the neck defect. The second horizontal (labeled 2 in Fig. 3), which passes through the two ischial tuberosities, creates another angle equal to the HEA (angle 4 + angle 5 = angle 3).
- The angle of the planned correction (angle 5 in Fig. 3) is calculated by subtracting 16° (angle 4, calculated from biomechanical analysis of the forces acting on the hip joint) from the HEA (angle 3); (note that angle 5 = angle 3 − angle 4).
- The correction (osteotomy) angle (angle 5 in Fig. 3) can then be drawn in the planned osteotomy site (angle 6 in Fig. 3). The* upper green line* in Fig. 3 is drawn in the intertrochanteric area tangent to the femoral neck defect and parallel to an imaginary line (7 in Fig. 3) bisecting the neck of the femur. The second line of the osteotomy is drawn at the calculated angle of correction from the first line.

### Operative technique

**Fig. 4:**
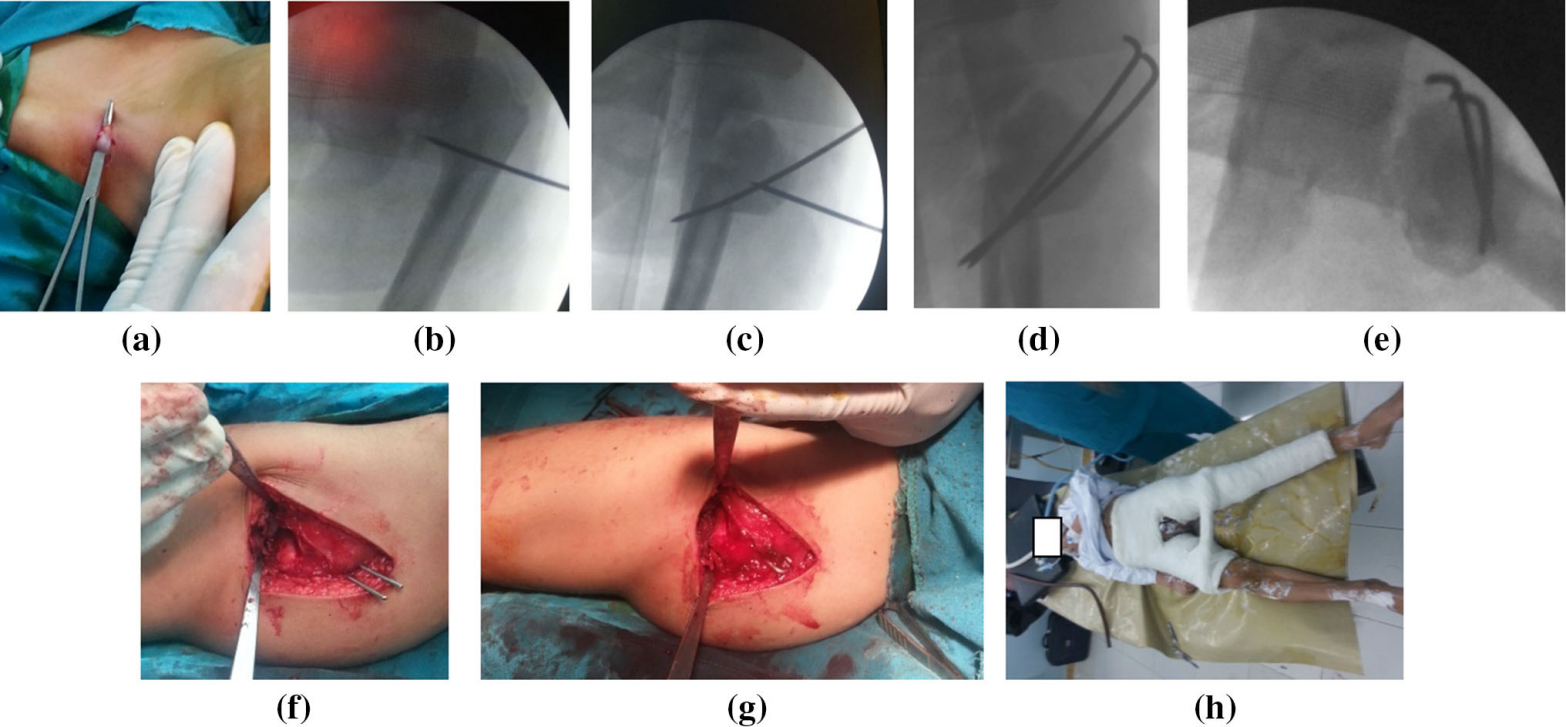
Summary of the surgical steps. **a** Adductor tenotomy. **b** Use of a percutaneous K wire under image-intensifier guidance to identify the appropriate osteotomy site. **c** A joystick wire was inserted in the trochanteric segment to aid manipulation and close the osteotomy site, and the first transfixing wire was directed from a superolateral to an inferomedial direction and engaged the medial cortex. **d, e** The two transfixing wires in their proper positions securing the osteotomy site in AP and lateral views. **f** No stripping was performed around the trochanteric area and neck of the femur, which allowed marked soft-tissue preservation. **g** The wires were bent and the soft-tissue sleeve around the osteotomy site was repaired. **h** A protective hip spica was applied

The procedure was performed in all cases under general anesthesia on a standard radiolucent operative table, with no traction needed. A very simple and important step—adductor tenotomy (Fig. 4a)—was carried out before approaching the femur and performing the osteotomy. Adductor tenotomy facilitates the closure of the osteotomy after wedge removal by allowing more abduction of the femur distal to the osteotomy site. The level of the planned osteotomy was determined percutaneously using the image intensifier (Fig. 4b) to minimize wound size. The proximal femur was exposed through a standard lateral approach, with no stripping of the proximal femur above the level of the trochanteric flare or exposure of the neck of the femur.

An incomplete V-shaped osteotomy was implemented as planned preoperatively using a power saw with preservation of a medial bony hinge at the apex of the osteotomy to be completed manually like osteoclasis. After wedge removal, the osteotomy site was closed by abducting the lower limb and manipulating the proximal fragment with the aid of a strong wire inserted as a joystick helping manipulation of this short segment producing the required correction. Now, the first transfixing K wire was inserted from proximal lateral direction pointing at and engaging the medial or posterior cortex of distal fragment at least 2 cm distal to the osteotomy site (Fig. 4c) and a second wire—at least—was inserted and checked with the image intensifier (Fig. 4d, e).

The number of transfixing wires and their diameters were determined by the size of the proximal fragment and the stability of the closed osteotomy site. No trochanteric apophysiodesis or trochanteric advancement was needed in any of the studied cases. A very important intraoperative sign of a good correction is the restoration of equal lower limb lengths in unilateral cases.

There was excellent preservation of the soft-tissue sleeve around the proximal femur, with complete avoidance and preservation of the neck of the femur and physeal plate (Fig. 4f, g). After proper hemostasis and closure of the wound without the use of a suction drain, a hip spica cast was applied (Fig. 4h).

### Postoperative evaluation and follow-up

**Fig. 5:**
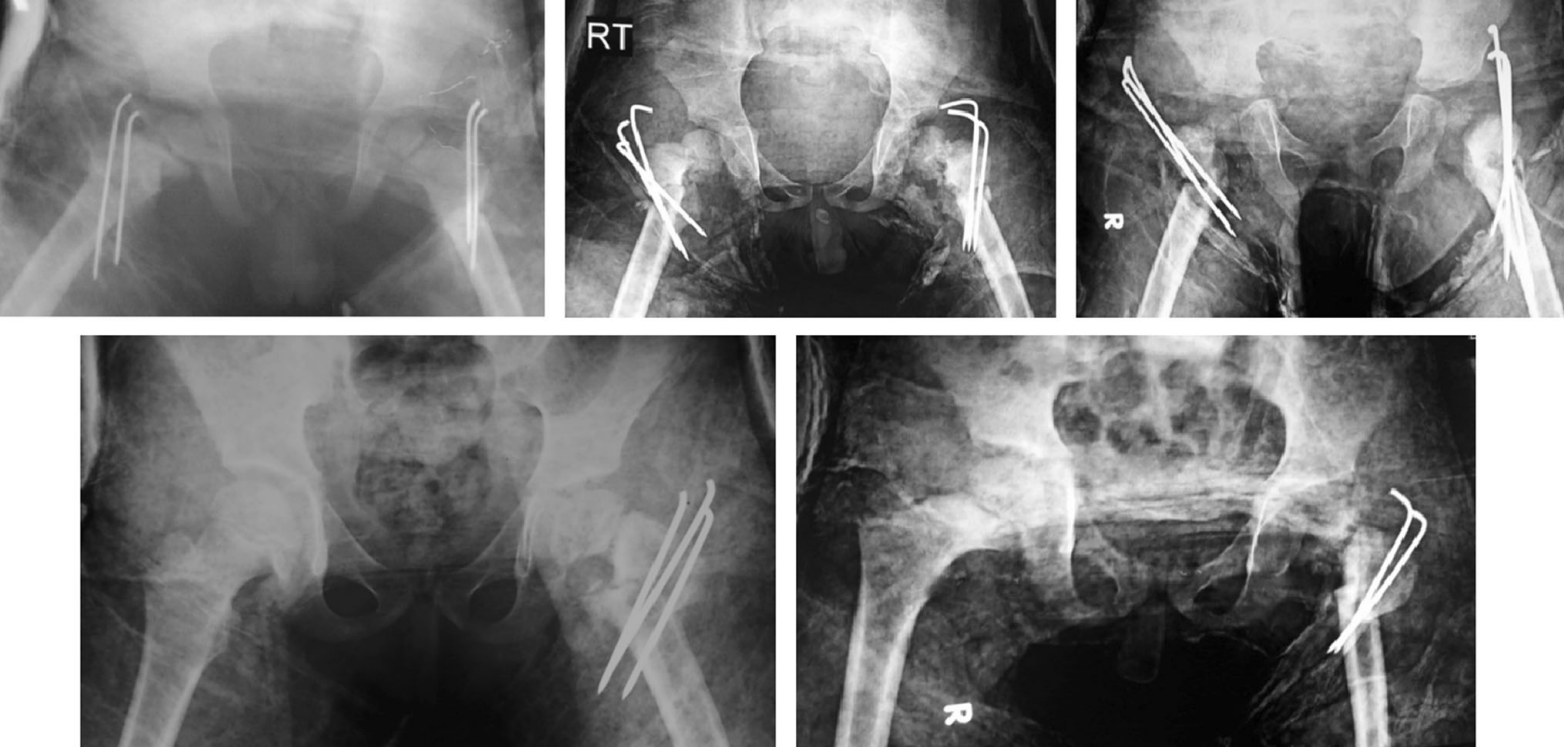
Immediate-postoperative X-rays of the hip spica casts, showing the correction achieved and the osteotomy fixation

There were no specific postoperative instructions for the patients, although the need for good hygiene and regular follow-up visits was emphasized. A postoperative X-ray was performed to evaluate the correction (Fig. 5). Patients were discharged from hospital the day after surgery and a broad-spectrum oral antibiotic and a simple analgesic were prescribed till the first follow-up visit 1 week after discharge for wound assessment (achieved via a small window in the spica cast). They then visited regularly at 2-week intervals for the first 3 months after the operation, monthly for the next 6 months, and yearly until the last follow-up.

The spica casts were removed once there was reliable callus formation in the follow-up X-rays (usually between postoperative weeks 5 and 7), and the patients were then allowed gradual weight bearing and both active and passive ranges of hip-joint movement. When the callus was mature and the osteotomy site was completely consolidated, the wires could be safely removed with no fear of deformation or late secondary displacement.

### Statistical analysis

Statistical analysis was performed using SPSS ver. 19.0 (SPSS Inc., Chicago, IL, USA). Statistical analysis was done using the two-tailed Student’s *t* test, and *p* < 0.05 was considered to indicate statistical significance.

## Results

The postoperative follow-up period ranged from 14 to 102 months, with an average duration of 33.3 ± 27.7 months. Clinically, there was a statistically significant (*p* < 0.001) improvement in the mean postoperative Iowa Hip Score at the last follow-up (95.06 ± 2.6) compared to the mean preoperative score (57.4 ± 3.6) (Table 1). Correction of LLD in unilateral cases, improved gait, and improved range of hip motion (especially abduction and easier ablution) were of paramount importance to parents as good indicators of successful surgery.

**Fig. 6:**
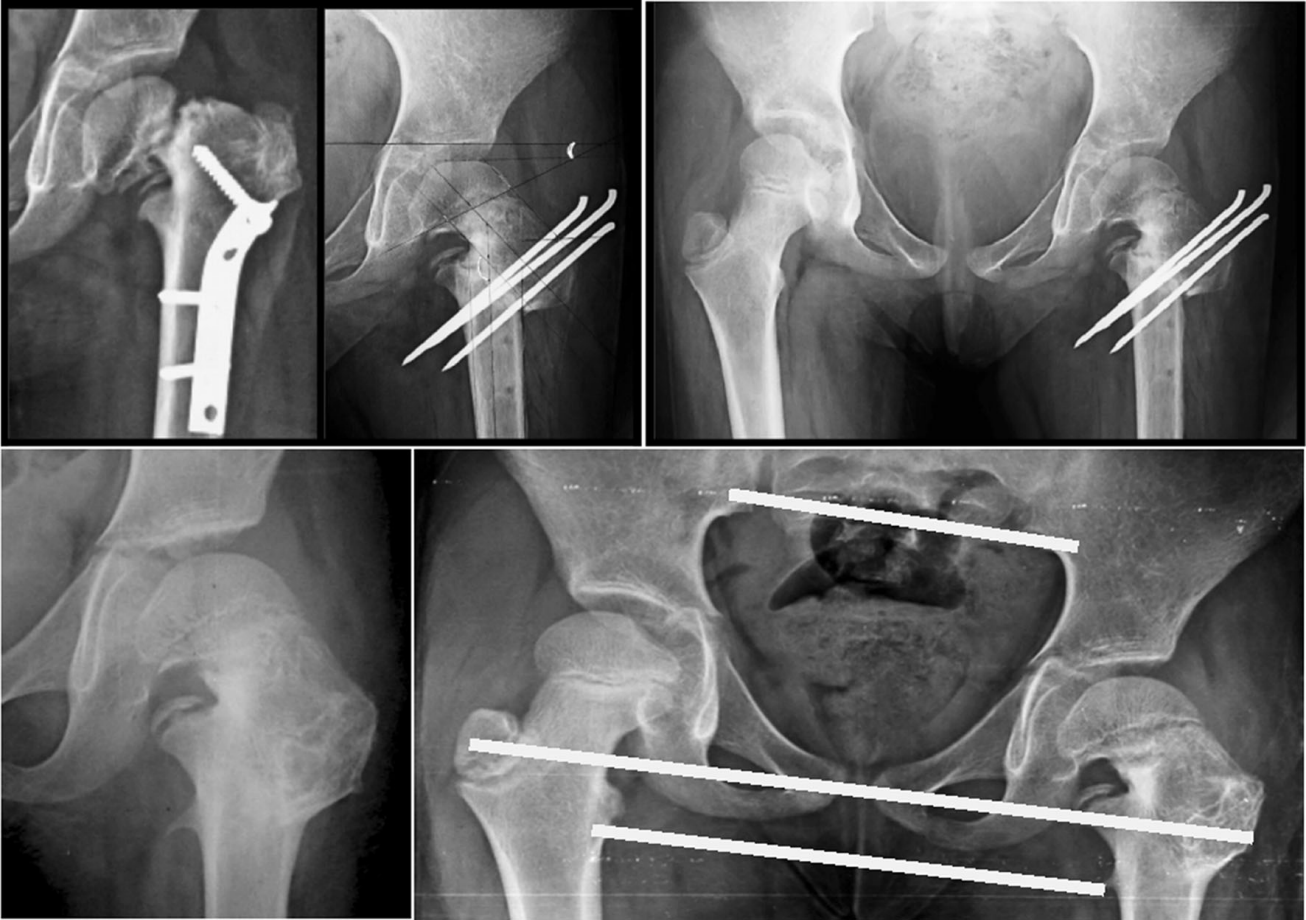
Revision osteotomy for residual coxa vara after a previous osteotomy that involved fixation with a plate. Revision osteotomy led to near-normal HEA and FNSA. The osteotomy healed completely, the femoral neck defect ossified, and the wires were removed. There was, however, incomplete correction of LLD, with about 1 cm of shortening observed

Two cases (both affected unilaterally) had LLD preoperatively; the LLD was corrected in one case and still present in the other. The latter case involved a revision surgery for a residual coxa vara after a previous valgus osteotomy fixed with a plate and screws 11 months before presentation. In that case, there was LLD of about 1 cm due to a subtracted bony wedge after the second osteotomy, resulting in residual shortening of the operated side; the patient tilted their pelvis slightly while walking to compensate (Fig. 6).

The osteotomies were performed in the metaphyseal, highly cancellous, proximal part of the femur characterized by high healing potential, and were completely united by an average of 11.7 ± 2.2 weeks (range: 9–15 weeks) without losing the correction achieved in any of the cases studied. The union time was not affected by the number of transfixing wires used, whether the coxa vara was unilateral or bilateral, or the size of the wedge removed. The wires could be safely removed with no fear of deformation or late secondary displacement once the callus was mature, but wires were not removed earlier than 6 months postoperatively in any of the cases.

The rationale for delaying the removal of wires until at least 6 months postoperatively—irrespective of whether there was full consolidation and callus maturation at the osteotomy site—was to allow the patients sufficient time to restore hip range and abductor power before they suffered a second operative trauma during wire removal. The wires were not removed in one patient (two cases) who refused early wire removal after complete union of the osteotomy, while the wires were removed in the other 14 cases at 6–10 months postoperatively (mean: 7.3 ± 1.4 months).

**Fig. 7:**
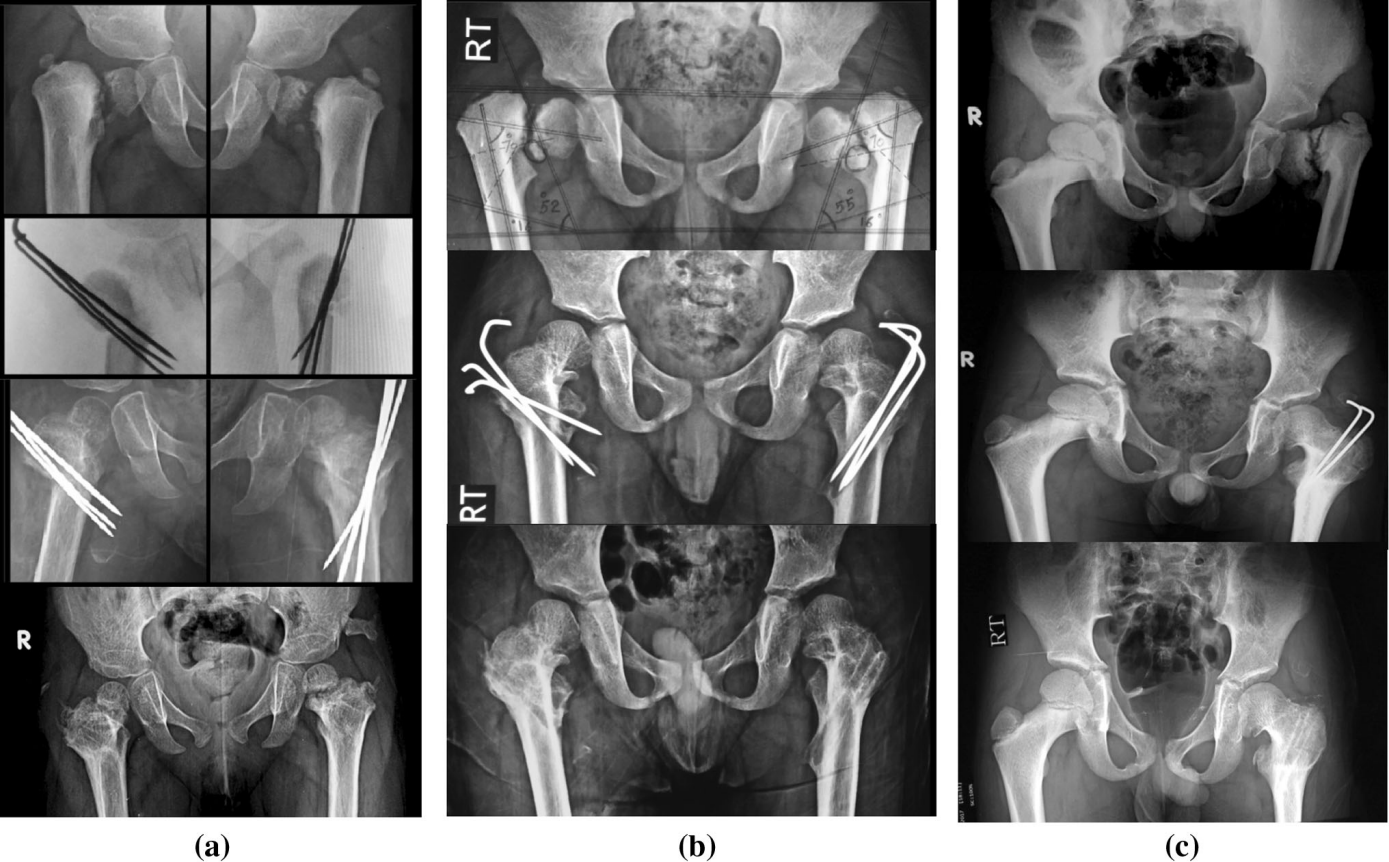
Radiological results for three different cases in the study. **a** Good radiological results with satisfactory restoration of HEA and FNSA in a 2-year-old female with bilateral developmental coxa vara after removal of the transfixing wires. **b** Good radiological results with satisfactory restoration of HEA and FNSA in a 4-year-old male with bilateral developmental coxa vara after removal of the transfixing wires. **c** Good radiological results (apart from premature closure of the proximal femoral epiphysis) with satisfactory restoration of HEA and FNSA in a 6-year-old male with unilateral atypical developmental coxa vara after removal of the transfixing wires

There were statistically significant improvements (*p* < 0.001) in the early postoperative HEA and FNSA compared to the preoperative angles. The mean HEA was corrected from 81.6 ± 8.2° (pre-op.) to 25.7 ± 2.5° (early post-op.), and the mean FNSA was improved from 89.7 ± 11.7° (pre-op.) to 137.3 ± 3.7° (early post-op.). The improvements in both the HEA and FNSA continued to be statistically significant (*p* < 0.001) at the last follow-up (24.3 ± 3.5° and 138.6 ± 3.5°, respectively) when compared to the preoperative values. Although progressive improvements of these angles—that could be attributed to the correction of the mechanical loading conditions and improved orientation of the growth plate—were reported in some cases with longer follow-up periods (Table 1), the overall early post-operative correction angles were statistically non-significantly different from the angles at the final follow-up. There was also progressive ossification of the neck defect in the 14 cases with developmental coxa vara (Fig. 7).

**Fig. 8:**
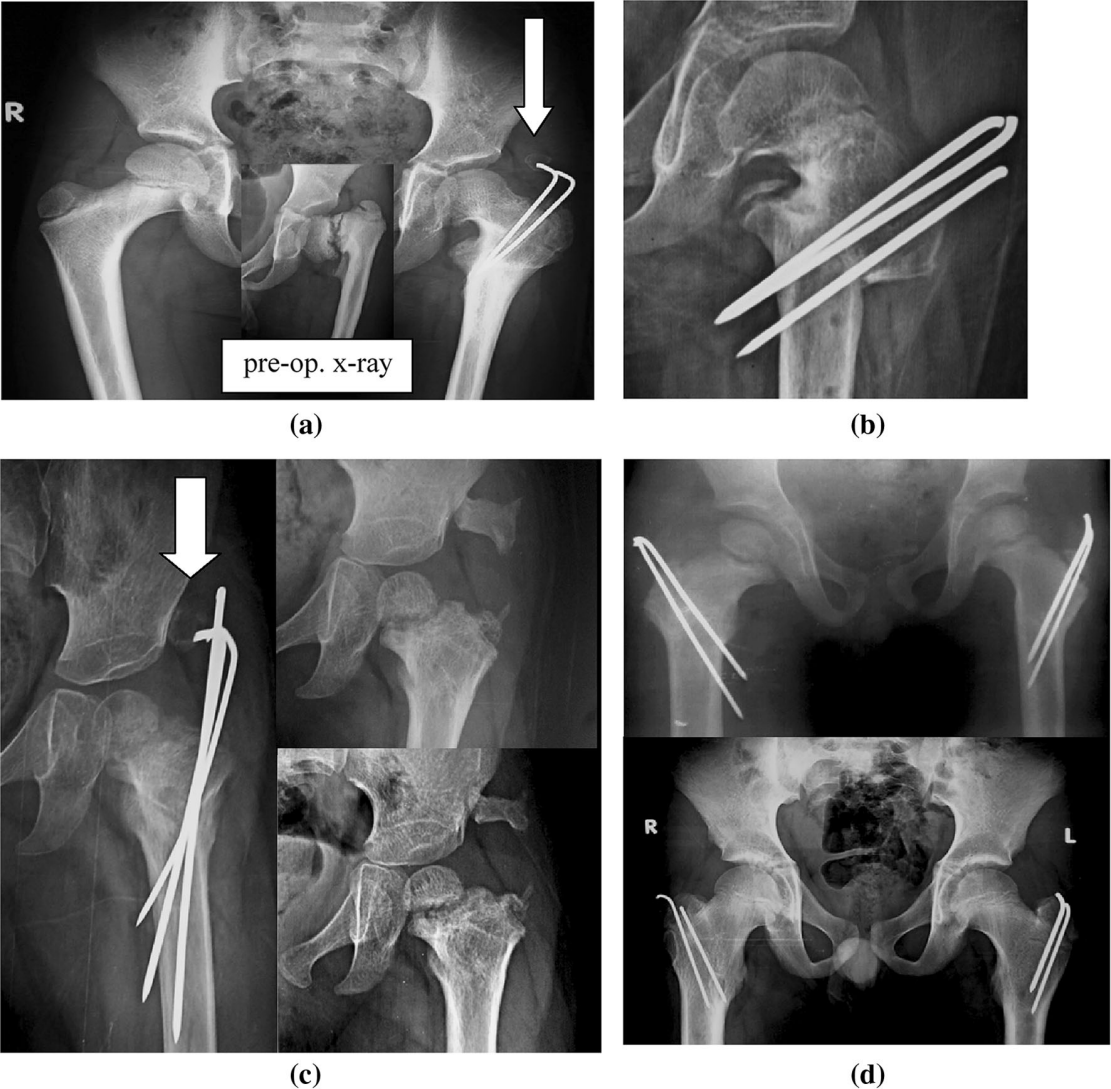
Radiological findings for some of the cases studied. **a** Premature proximal physeal closure and medial displacement of the distal fragment after osteotomy. The *white arrow* points to a hazy bony shadow associated with the ends of the wires in the gluteal region. **b** Marked medial displacement of the distal fragment after osteotomy in a revision case after a previous osteotomy fixed with plate and screws. **c** A hazy bony shadow associated with the ends of the wires in the gluteal region. This became mature bony mass in the abductors, which had no significant clinical implications and progressively reduced in size with maturation. **d** Sunken wires with growth of the patient for 8.5 years postoperatively, showing remodeling of the acetabulum and proximal femur

Although the procedure decreases soft-tissue stripping and completely avoids the femoral neck and physeal plate, premature physeal closure occurred unexpectedly in one case (Fig. 8a, corresponding to the case presented in Fig. 1d). This can be explained by the marked severity of the deformity in this case of developmental coxa vara, where there was an atypical defect affecting both the superior (lateral) and inferior (medial) parts of the femoral neck. Medialization of the distal fragment after osteotomy (Fig. 8b) occurred in three cases (two primary cases and one revision case), but there were no significant clinical implications of the medialization during the follow-up period.

Heterotopic bone formation in the gluteal region occurred in two cases. Bone formation started as a hazy shadow in the abductors, which could have been due to muscle irritation by the wires. This gradually matured into a well-formed bony mass that had no significant clinical implications (Fig. 8a, c) and gradually decreased in size over the subsequent follow-up. The wires were sunken in the trochanteric apophysis in one patient (two cases; Fig. 8d) who refused early wire removal after complete union of the osteotomy but suffered no related complications throughout 8.5 years of follow-up.

None of the cases had developed avascular necrosis, complications related to this method of fixation, recurrence of the hip deformity, or genu valgum deformity of the knees by the last follow-up; this was even true of the three cases in which medialization of the distal fragment occurred, as remodeling of the proximal femur in this age group is excellent, permitting progressive correction and restoration of alignment with no residual radiological or clinical effects.

## Discussion

Theoretically, surgical correction of the vertically oriented femoral physis to a relatively horizontal position with a Hilgenreiner epiphyseal angle (HEA) of less than 38° will normalize the biomechanical forces, converting the poorly tolerated shear forces to more physiological and desirable compressive forces. This leads to greater protection of the physis and stimulates proper ossification of the femoral neck defect, reducing the risk of recurrence of coxa vara whatever the etiology of the deformity and the age of the patient [3, 5].

Different techniques based on the same surgical principles but utilizing different implants for internal or external fixation of either intertrochanteric or subtrochanteric valgus osteotomies have been described for the surgical correction of coxa vara. The surgical technique most commonly used to achieve correction is the laterally based, intertrochanteric Y-shaped wedge osteotomy described by Pauwels [6], considered to be state of the art in the surgical management of infantile coxa vara [2]. Pauwels’s Y-shaped osteotomy is performed between the lesser and greater trochanters and perpendicular to the femoral shaft. It corrects the neck–shaft angle but the femoral shaft is positioned medially. However, it compromises the hip joint’s integrity, potentially endangering the nourishing vessels of the femoral head and thus increasing the risk of avascular necrosis [2]. Medial displacement in such patients should be strongly avoided as it exacerbates the loading of the distal femoral physis and the lateral compartment of the knee in patients with knee deformity (genu valgum), which is commonly also seen in cases of coxa vara [7].

Borden et al. [8] presented a subtrochanteric valgization osteotomy, which is a perpendicular osteotomy in the subtrochanteric region just distal to the lesser trochanter. They introduced the concept of end-to-side valgization osteotomy in cases of infantile coxa vara, which presents only a minimal risk of head necrosis due to its extra-articular approach and lack of medialization of the distal femoral shaft. It also requires only one osteotomy with easier insertion of the angled plate, making it less technically demanding than the standard intertrochanteric technique [2]. Percutaneous subtrochanteric osteotomy with external fixation has been reported as an alternative to internal fixation [9], but that study included only limited follow-up.

**Fig. 9:**
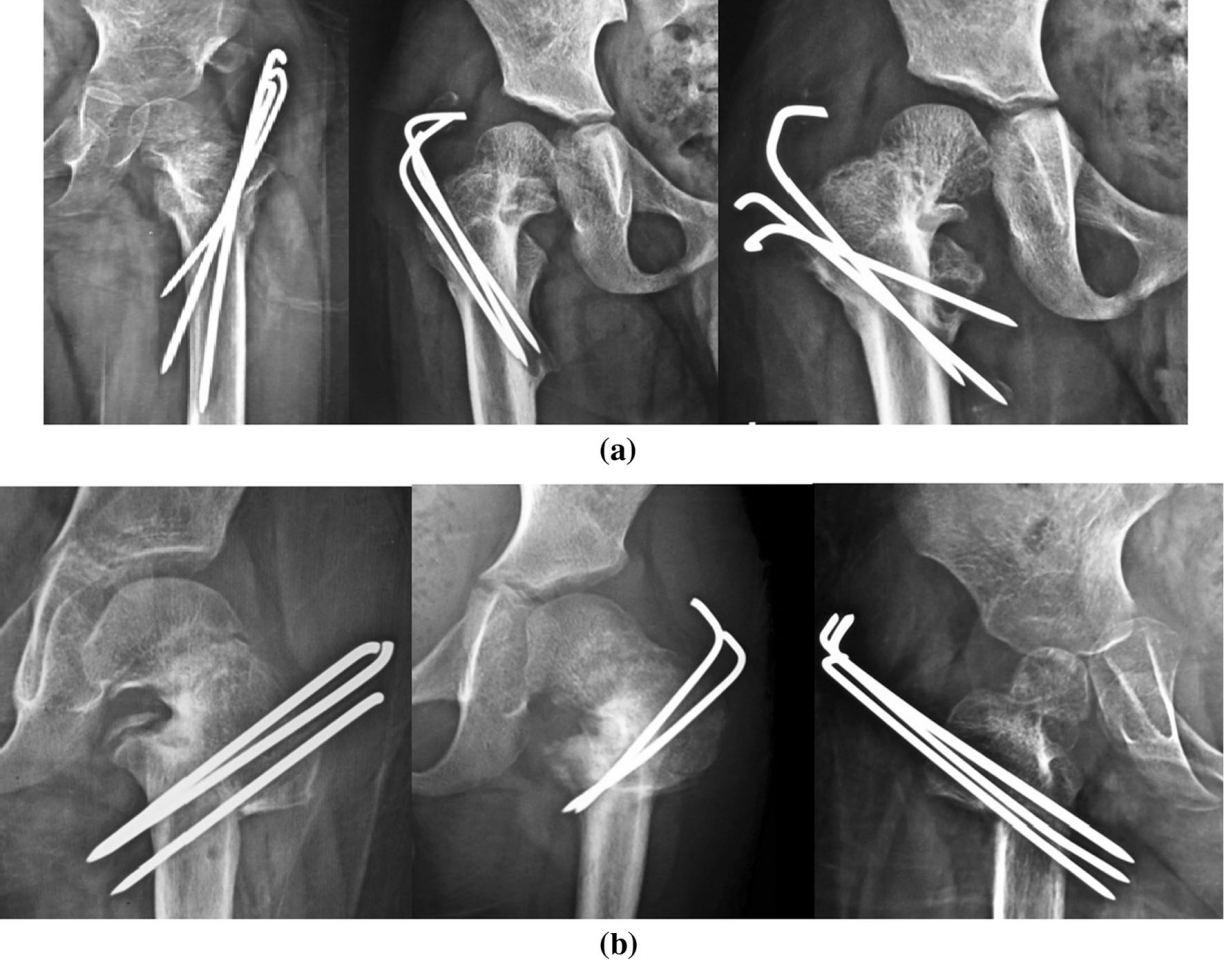
Alignment of the femur after corrective osteotomy and fixation. **a** Proper alignment with no medialization of the distal fragment. **b** Medialization of the distal fragment

**Fig. 10:**
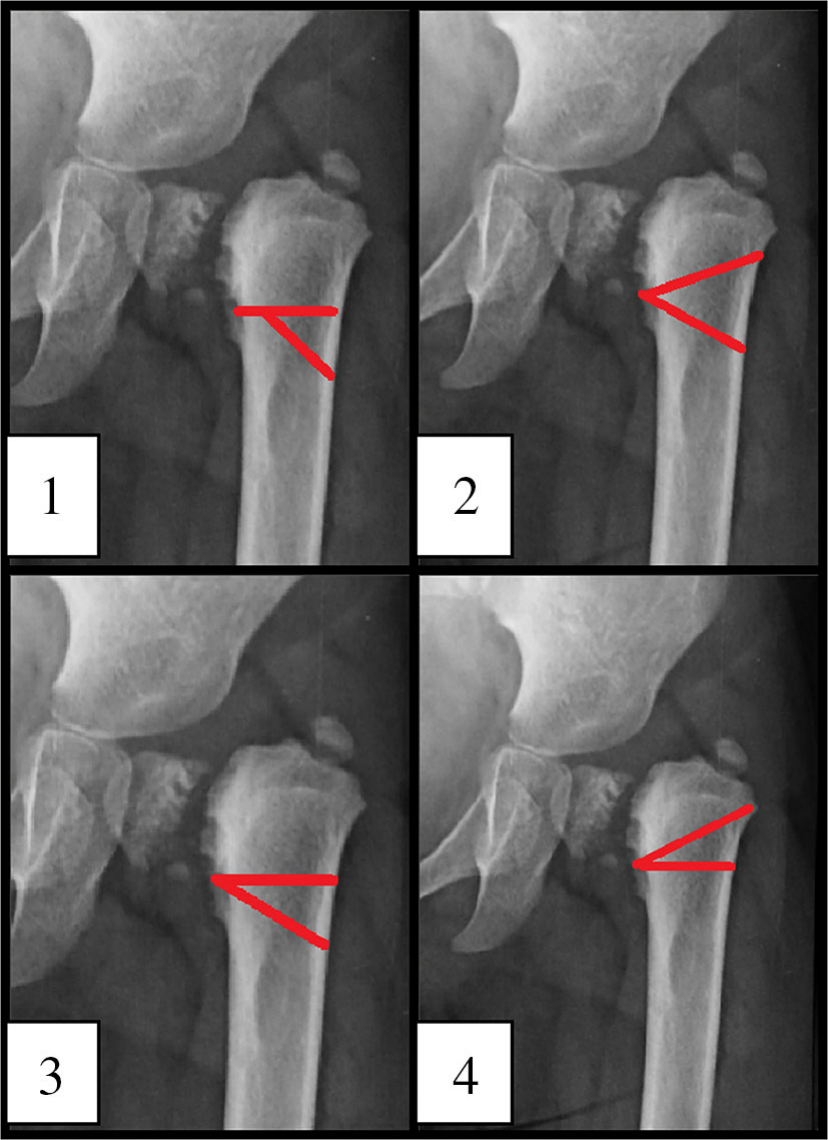
Diagrammatic presentation of various possible shapes of intertrochanteric osteotomy. *1* Classical Y-shaped osteotomy. *2* V-shaped osteotomy with both arms of the osteotomy oblique. *3*, *4* V-shaped osteotomy with a horizontal proximal or distal arm and the other arm oblique

In the present study, proper correction of the FNSA and HEA was achieved in all of the studied cases. There was no medialization of the distal fragment (Fig. 9a) after osteotomy in 13 cases, while there was medialization of the distal fragment (Fig. 9b) after osteotomy in three cases (two primary cases and one revision case). In contrast to the classical technique (Pauwels’s Y-shaped osteotomy; Fig. 10-1), the osteotomy performed in this work was a V-shaped, intertrochanteric osteotomy that—like Pauwels’s osteotomy—corrected the neck–shaft angle appropriately, but with less medialization of the distal fragment after osteotomy.

It was noted—retrospectively—that the best proportion and coaptation between the proximal and distal bone fragments after wedge removal occurred with both the upper and lower arms of the osteotomy oblique (Fig. 10-2), while medialization occurred in cases where either the proximal or the distal arm of the osteotomy was horizontal (Fig. 10-3, -4).

**Fig. 11:**
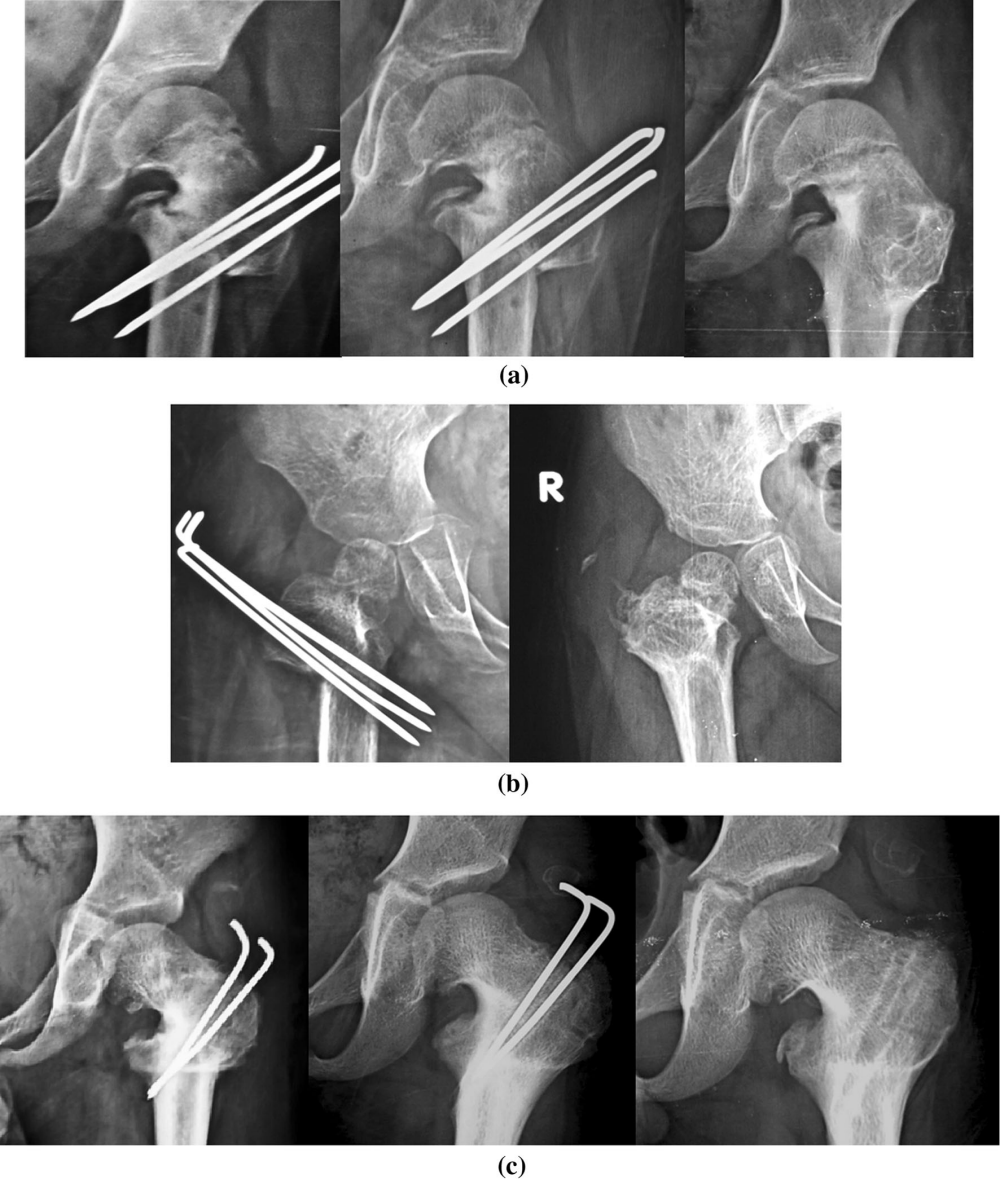
Remodeling improved the overall alignment of the operated side during follow-up. **a** Progressive remodeling had effectively corrected the medial displacement of the distal fragment after 27 months postoperatively. **b** Progressive remodeling had effectively corrected the medial displacement of the distal fragment after 12 months postoperatively. **c** Progressive remodeling had effectively corrected the medial displacement of the distal fragment after 22 months postoperatively

Although remodeling can improve the alignment during the follow-up period (Fig. 11), rendering medialization a benign issue, Medialization of the distal fragment should be avoided to decrease the incidence of post-operative genu valgus which is a common finding with severe pre-operative coxa vara or after proximal femoral valgus osteotomies if medialization occurred. Prober alignment of the osteotomy ends with no—or minimal—medialization facilitates transfixing wiring with better bone hold and less metal prominence under the skin.

It is reported in the literature that there is limited choice when selecting an implant that allows secure fixation of such a small proximal fragment of bone while achieving stable fixation of the osteotomy without violating the proximal femoral growth plate. Angled blade plates have been the standard devices used for fixation of proximal femoral osteotomies in children [10]. Nevertheless, there are problems with this technique; for example, the plate is not a suitable size for some children and considerable technical skill is required for its insertion. Experimental studies have indicated that the tension-band wiring fixation of the proximal femur, as proposed by Pauwels and adapted by Weber, could overcome these limitations and provide simple and stable fixation [11, 12]. External fixators have been used as an alternative to internal fixation in percutaneous subtrochanteric osteotomy, but the reported results include only limited follow-up [9], and there are potential risks of pin tract infection and loosening.

In this study, the selected method of fixation was to apply transfixing K wires protected with a hip spica cast for an average duration of 6 weeks. Transfixing K wires are versatile, malleable tools for stable fixation, even with larger angles of correction that cannot usually be addressed using plates without contouring them to such an extent that the plate strength may be comprised. The application of transfixing K wires requires less dissection, thus allowing the soft tissues around the proximal femur above the level of the trochanteric flare to be largely preserved and decreasing the healing time, it also completely avoids violating the femoral neck affording marked protection to the precious growth plate.

In this study, the only disadvantage of transfixing wires was excessive protrusion of the wires from the medial cortex, leading to a risk of injury to the medially located vessels. Meticulous insertion and application of the transfixing wires with just few millimeters of protrusion from the medial cortex of the proximal femur is recommended and can be assured through the use of an image intensifier.

Using transfixing K wires without a tension band made the procedure easier and faster, with no fear of premature closure of the trochanteric apophysis. As they are hidden within the wound and protected by the cast, transfixing K wires can give superior results to external fixators, with no risk of pin tract infection or loosening. The hip spica used to protect against displacement at the osteotomy site was not unique or specific for transfixing wires, but it was used in some studies for several weeks after surgery, even when that involved fixation by plates and screws [3, 8, 13].

The results of this study were comparable to—and, to some extent, better than—the results of other studies concerning coxa vara, regardless of the etiological cause, osteotomy site, and mode of fixation. The mean HEA had been corrected from 81.7 ± 2.2° to 24.3 ± 3.5° by the last follow-up and the mean FNSA had improved from 86.9 ± 4.2° to 138.6 ± 3.5°. In a study carried out by Cordes et al. [5], which included 18 cases of coxa vara in 14 patients with various etiologies, the mean postoperative correction of the FNSA was 141° and that of HEA was 29°. Another study, performed by Desai and Johnson [14], included 20 cases of coxa vara in 12 patients. Their mean postoperative correction of the FNSA was 136° and that of the HEA was 30°. In a study by Hefny et al. [15] of the use of external fixators to treat coxa vara in 15 cases in 13 patients, the mean postoperative correction of the FNSA was 133° and the HEA was 34°.

In a more recent study by Elzohairy and Khairy [13], in which a T plate was employed in the fixation of intertrochanteric valgus osteotomy to treat 18 cases of developmental coxa vara in 18 patients, the mean postoperative correction of the FNSA was 129.9° and that of the HEA was 27.8.

The literature reports recurrence rates of between 30 and 70% [16]. Carroll et al. [3] said that correction of the HEA to <38° lowers the recurrence rate to about 5% of the corrected cases. Carroll et al. [3] concluded that the neck–shaft angle was not a reliable indicator of appropriate correction or outcome, and they reported that recurrence was mainly due to the loss of the correction angle resulting from weak fixation. This explains why there was no recurrence of deformity in any of the cases studied right up to the last follow-up, as the mean correction of the HEA was 24.3 ± 3.5°, which is quite close to the normal (anatomic) value of HEA: about 22° [3]. It also indicates that using only transfixing wires with a protective cast is an efficient and reliable method of fixation for these osteotomies, as it prevents any postoperative displacement and loss of the correction angle.

Intraoperatively, it was found that patients with unilateral coxa vara gain length after the corrective osteotomy, with the reported preoperative LLD eliminated. In bilateral cases, there was lengthening of the first limb to be operated by about 1–2 cm in comparison to the other side after the corrective osteotomy, although patients had no LLD preoperatively. Such deformity was corrected and led to overall lengthening of both sides. In other words, although a closing wedge (subtraction) osteotomy was performed, the overall length of each limb was increased after proper redirection of the femoral neck and correction of the deformity.

The relatively short mean follow-up period in such a small number of cases, the lack of alternatives (different types of osteotomy and different fixation methods) for use as a control group, and the absence of any biomechanical lab studies comparing different fixation methods under different loading conditions are limitations of this study. However, these limitations do not appear to undermine the results achieved in this study. Further research correlating different variables such as the age of the patient, the etiology and severity of their deformity, the fixation method used, the radiological healing time, the functional results, and complications is strongly recommended.

In conclusion, transfixing wires protected in a hip spica cast represent a simple, easy, and reliable fixation method for valgus osteotomies performed to correct different varieties of pediatic coxa vara (even revision cases) with different degrees of severity. It assures stable fixation and rapid healing of the osteotomy without loss of the achieved correction. It completely avoids the femoral neck affording marked protection to the growth plate.
